# Two species? – Limits of the species concepts in the pygmy grasshoppers of the *Tetrix bipunctata* complex (Orthoptera, Tetrigidae)

**DOI:** 10.3897/zookeys.1043.68316

**Published:** 2021-06-11

**Authors:** Valentin Moser, Hannes Baur, Arne W. Lehmann, Gerlind U. C. Lehmann

**Affiliations:** 1 Ochsengasse 66, 4123 Allschwil, Switzerland Unaffiliated Allschwil Switzerland; 2 Department of Invertebrates, Natural History Museum Bern, Bernastrasse 15, 3005 Bern, Switzerland Natural History Museum Bern Bern Switzerland; 3 Institute of Ecology and Evolution, University of Bern, Baltzerstrasse 6, 3012 Bern, Switzerland University of Bern Bern Switzerland; 4 Specialist Interest Group Tetrigidae (SIGTET), Friedensallee 37, 14532 Stahnsdorf, Germany Specialist Interest Group Tetrigidae Stahnsdorf Germany; 5 Department of Biology, Evolutionary Ecology, Humboldt University Berlin, Invalidenstrasse 110, 10115 Berlin, Germany Humboldt University Berlin Berlin Germany

**Keywords:** Allometry, integrative taxonomy, morphometry, Orthoptera, species delimitation, Tetrigidae, *
Tetrix
*

## Abstract

Today, integrative taxonomy is often considered the gold standard when it comes to species recognition and delimitation. Using the *Tetrix bipunctata* complex, we here present a case where even integrative taxonomy may reach its limits. The *Tetrix bipunctata* complex consists of two morphs, *bipunctata* and *kraussi*, which are easily distinguished by a single character, the length of the hind wing. Both morphs are widely distributed in Europe and reported to occur over a large area in sympatry, where they occasionally may live also in syntopy. The pattern has led to disparate classifications, as on the one extreme, the morphs were treated merely as forms or subspecies of a single species, on the other, as separate species. For this paper, we re-visited the morphology by using multivariate ratio analysis (MRA) of 17 distance measurements, checked the distributional data based on verified specimens and examined micro-habitat use. We were able to confirm that hind wing length is, indeed, the only morphological difference between *bipunctata* and *kraussi*. We were also able to exclude a mere allometric scaling. The morphs are, furthermore, largely sympatrically distributed, with syntopy occurring regularly. However, a microhabitat niche difference can be observed. Ecological measurements in a shared habitat confirm that *kraussi* prefers a drier and hotter microhabitat, which possibly also explains the generally lower altitudinal distribution. Based on these results, we can exclude classification as subspecies, but the taxonomic classification as species remains unclear. Even with different approaches to classify the *Tetrix bipunctata* complex, this case is, therefore, not settled. We recommend continuing to record *kraussi* and *bipunctata* separately.

## Introduction

Species concepts shape the way we see an individual from a given population. Species are the fundamental unit in evolutionary biology (Coyne and Orr 2004) and it is, therefore, important to apply the species status to the best of our current knowledge (Sites and Marshall 2004). Species discovery and description remain a core priority of taxonomic research and critical reflection of current practice is called for (Yeates et al. 2011). Traditionally, species were mostly based on morphological characters. With the advance of technology and easier access to genomes, species classification criteria have diversified (Wägele 2005; Zachos 2016). To generalise species classification and comparability, attributes, such as morphology, genetics, behaviour and ecology are treated as evidence (Dayrat 2005; Will et al. 2005; De Queiroz 2007; Yeates et al. 2011). However, there are still cases where the assignment is difficult, even when using a variety of data. Here, we present such a case in the Pygmy Grasshopper of the family Tetrigidae.

**Figure 1.**
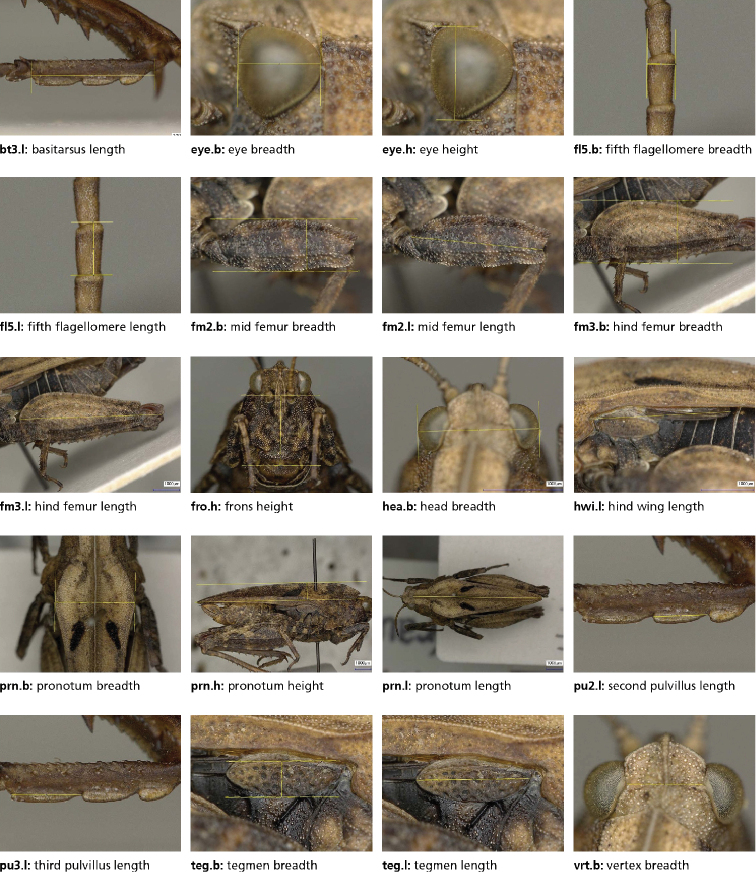
The 20 characters measured on 273 females of *Tetrix bipunctata* and *kraussi*. Measurements indicated by yellow lines. In all cases, a single photo was taken with reference points exactly placed in the focal plane. For character definitions, see Table 1.

The *Tetrix bipunctata* complex is an intriguing case: *T. bipunctata* (Linnaeus, 1758) and *T. kraussi* Saulcy, 1888 (see Evenhuis 2002 for year of publication) are two widely distributed European Orthoptera of the family Tetrigidae. They are considered morphologically very similar, except for a striking hind wing dimorphism. In the morph *bipunctata*, the hind wing is said to be at least 2.5 times as long as the length of the tegmen, whereas in the morph *kraussi*, it is only about twice as long as the tegmen (sometimes also called tegmentulum, Fig. 1) (Fischer 1948; Schulte 2003; Baur et al. 2006; Lehmann and Landeck 2011; Sardet et al. 2015a).

The status of the two morphs has always been controversial. Fischer (1948) recognised ecological differences and suggested to treat them as species, but this view was later challenged. For example, the morphs were treated only as infrasubspecific taxa by Kevan (1953) and Harz (1957, 1975), but also as subspecies by Nadig (1991). Based on several syntopic occurrences (Schulte 2003), Lehmann (2004) suggested to raise the morphs to species status, a view that has since been widely adopted (Baur et al. 2006; Default and Morichon 2015; Sardet et al. 2015b; Zuna-Kratky et al. 2017; Willemse et al. 2018; Cigliano et al. 2021), with some exceptions (Wranik et al. 2008; Pfeifer et al. 2011; Massa et al. 2012; Bellmann et al. 2019; Fischer et al. 2020)

Some authors have suggested that there are further morphological characters besides the hind wing that would allow us to distinguish the two morphs. Koch and Meineke (in Schulte 2003) state that, not only the length of the hind wing, but also the extent of the tegmen and the height of the pronotum significantly differ between the two morphs. Schulte (2003) used a sex-specific ratio of hind wing length to pronotum length to determine the morphs. Furthermore, it was suggested that *bipunctata* is, on average, slightly larger and the pronotum more strongly arched (e.g. Baur et al. 2006).

No genetic differences have been found so far, as the two morphs form a single cluster when compared using COI barcoding (Hawlitschek et al. 2017).

In this study, we examine the morphs *bipunctata* and *kraussi* and discuss their status, based on new data from: (1) multivariate morphometry, (2) biogeography in Central Europe and (3) microhabitat niche use in syntopy.

1) Concerning morphological characters, we address the following questions:

– Are further characters – besides wing length – important for the separation of bipunctata and kraussi and to what extent? Some authors claim that body proportions seem to differ; however, nobody has ever tried to quantify those traits.
– What are the best shape characters for separating bipunctata and kraussi? As mentioned before, so far only a single ratio, hind wing length to tegmen length (either by taking into account the entire hind wing length or just the part projecting beyond the tegmen), has been used regularly. A morphometric analysis thus might reveal some more reliable ratios.
– Despite the evidence for two distinct morphs (Fischer 1948; Schulte 2003), specimens with intermediate wing ratios have been reported by Nadig (1991). Therefore, we re-examined Nadig’s collection including the specimens in question.
– How much allometry is present? Size-dependent variation in the adult stage (static allometry, see Gould 1966; Klingenberg 2008, 2016; Anichini et al. 2017; Rebrina et al. 2020) plays a major role in such investigations, but so far, it has been neglected in this complex. Here, we analyse which characters and character ratios correlate with body size.

2) Biogeography

Due to the uncertain taxonomic situation, the distribution is far from settled, as many authors have not differentiated between *bipunctata* and *kraussi.* Furthermore, a substantial number of misidentifications have been published for *Tetrigidae* (own results, compare Lehmann et al. 2017). To establish a firm database for the distribution, we studied specimens from European Museums, complemented by private collections. The material from six central European countries added up to 663 specimens. This allows us to analyse the distribution and especially the level of sympatry and even syntopy. Furthermore, we study the altitudinal range separately for *bipunctata* and *kraussi*.

3) Ecology of habitat use at a syntopic population in Brandenburg

The segregated distribution of *bipunctata* and *kraussi* is interpreted as an ecological separation (Fischer 1948; Lehmann 2004). To test for differential habitat use, we studied microhabitat niches in a syntopic population discovered in southern Brandenburg (Lehmann and Landeck 2011).

## Materials and methods

### Identification of specimens

Below, we consistently refer to the morphs as “*bipunctata*” and “*kraussi*” and treat them in the sense of operational taxonomic units. For the assignment of specimens to morphs, we adopted the identifications found on the labels in the Swiss collections. This was mainly the case for specimens in Nadig’s collection, also with respect to what he considered as intermediate specimens. In all other instances we followed current practice (Schulte 2003; Lehmann 2004; Baur et al. 2006) and calculated the ratio of the full hind wing length to tegmen length: ≥ 2.5 = *bipunctata*, < 2.5 = *kraussi* (corresponding to the ratio of the protruding part of hind wing length to tegmen length of ≥ 1.5 and < 1.5, respectively). The same threshold was applied for a very few specimens that had obviously been misidentified by Nadig. The assignment of specimens was done before we performed any of the analyses reported below. As mentioned in the Introduction, *bipunctata* and *kraussi* have traditionally been separated by this ratio, which is why we refer to it as the “standard ratio” below.

### 1) Morphometry

#### Character measurements

We measured 20 characters from all over the body to cover the most relevant variation in size and shape between *bipunctata* and *kraussi*. The selection of characters was based on Harz (1975), Devriese (1996), Tumbrinck (2014) and our own expertise. Characters are shown in Fig. 1, definitions being given in Table 1. An overview of the basic descriptive statistics for each measurement (in mm) and morph, as well as the sample sizes is given in Appendix 2. We base our morphometric study on females because they were available in larger numbers. A further strength of using females is their larger body size, making measurements easier and faster. The majority of specimens originated from the collection Nadig (in Muséum d’histoire naturelle, Geneva, Switzerland, MHNG), the rest consisting of older material collected by Baur (in coll. Nadig) and some specimens collected in 2015 (also in Naturhistorisches Museum Bern, Switzerland, NMBE). We included 273 females from various populations in Central Europe, mainly from the Alps and the Jura (Table 2).

**Table 1.** Abbreviation, name, definition and magnification (on Keyence digital microscope) of the 20 measurements used for the morphometric analyses of *Tetrix bipunctata* complex females. General morphology follows Lawrence et al. (1991) and the morphological terminology for pronotal carinae is adopted from Devriese (1996).

No.	Abbrev.	Character name	Character definition	Magnification
1	bt3.l	Basitarsus length	Length of basitarsus of hind tarsus, from proximal expansion to apex, outer aspect along ventral side	150
2	eye.b	Eye breadth	Greatest breadth of eye, lateral view	150
3	eye.h	Eye height	Greatest height of eye, lateral view	150
4	fl5.b	5^th^ flagellomere breadth	Greatest breadth of 5^th^ flagellomere, dorsal (inner) aspect	150
5	fl5.l	5^th^ flagellomere length	Greatest length of 5^th^ flagellomere, dorsal (inner) aspect	150
6	fm2.b	Mid-femur breadth	Greatest breadth of mid-femur, lateral view	100
7	fm2.l	Mid-femur length	Length of mid-femur, from proximal emargination of trochanter to emargination of knee, lateral view	100
8	fm3.b	Hind femur breadth	Greatest breadth of hind femur, lateral view	30
9	fm3.l	Hind femur length	Length of hind femur, from proximal edge to tip of knee disc, lateral view	30
10	fro.h	Frons height	Height of frons, from lower margin of clypeus to lower margin of eye orbit, frontal view	100
11	hea.b	Head breadth	Greatest breadth of head, dorsal view	100
12	hwi.l	Hind wing length	Length of hind wing, from proximal edge of tegmen to tip of hind wing, in situ. *Remark*: Very often, only the part protruding below the tegmen has been considered. Unfortunately, the measurement is then critically dependent on the position of the tegmen, which is often displaced relative to the hind wing. We, therefore, preferred the entire hind wing length, which can be measured rather more reliably	30
13	prn.b	Pronotum breadth	Greatest breadth of pronotum, dorsal view	30
14*	prn.h	Pronotum height	Greatest height of pronotum, from carina humeralis at level of proximal edge of tegmen to highest point of carina medialis, exact lateral view	30
15	prn.l	Pronotum length	Length of pronotum, from anterior margin to the tip of the posterior pronotal process, dorsal view along carina medialis	30
16*	pu2.l	2^nd^ pulvillus length	Length of 2^nd^ pulvillus on basitarsus of hind tarsus, from its proximal notch to distal notch, outer aspect	150
17*	pu3.l	3^rd^ pulvillus length	Length of 3^rd^ pulvillus on basitarsus of hind tarsus, from its proximal notch to distal notch, outer aspect	150
18	teg.b	Tegmen breadth	Greatest breadth of sclerotised part of tegmen, outer aspect	100
19	teg.l	Tegmen length	Length of fore wing, from proximal edge of tegmen to tip of fore wing, outer aspect	100
20	vrt.b	Vertex breadth	Shortest breadth of vertex, dorsal view. Together with head breath, this covers also potential differences in eye breath.	100

* Character omitted in morphometric analyses, see Appendix 1.

**Table 2.** Overview on *Tetrix bipunctata* complex populations (females only) included in the morphometric analyses. Most specimens are from the Nadig collection in MHNG.

Country	Population
AT	Kärnten
CH	BE Beatenberg
CH	BE/JU Jura
CH	GR Oberengadin
CH	GR Schams
CH	GR Unterengadin
CH	UR Urnerboden
DE	S-Bayern
DE	Schwarzwald
IT	Chiavenna
IT	Como
IT	Gardasee
IT	S-Tirol E/Mittenwald
IT	Trentino

Each character was photographed with a Keyence VHX 2000 digital microscope and a VH-Z20R/W zoom lens at different magnification, depending on the size of the body part (see Table 1). For most measurements, we ensured that the reference points were placed exactly in the focal plane. Only one character, pronotum height (prn.h), was exceptional in that the reference points were not exactly in the same focal distance; here also, just a single photo was necessary, because the depth of field was sufficiently large. Moser took the photographs and measured the distances using ImageJ v.1.49r (Schneider et al. 2012); body parts on the images were zoomed in 3–4 times before measuring. Three characters were eventually omitted from the morphometric analysis (explained in Appendix 1), because of strong individual variation (pronotum height) or wear (2^nd^ and 3^rd^ pulvillus length), so that the final data contained 17 characters.

#### Multivariate ratio analysis of the body measurements

For the data analysis, we applied multivariate ratio analysis (MRA) (Baur and Leuenberger 2011). MRA comprises several tools related to standard multivariate methods, such as principal component analysis (PCA) and linear discriminant analysis (LDA). Contrary to the normal application of these methods, MRA allows the interpretation of size and shape in a manner that is entirely consistent with the customary usage of body lengths and body ratios in taxonomy, for instance, in descriptions and diagnoses. Examples of the application of different MRA tools may be found in various papers (László et al. 2013; Baur et al. 2014; Ali et al. 2016; Huber and Schnitter 2020; Le et al. 2020; Selz et al. 2020). Here, we first calculated a general measure of size, “isosize”, which we obtained by calculating for each specimen the geometric mean of all measurements. We then performed a PCA on a data matrix, where we divided each value by isosize, thus entirely removing differences in isometric size. To distinguish this particular type of PCA from the usual one based on just log-transformed raw data (Jolicoeur 1963), we called it “shape PCA” below.

Very often shape correlates with size, which corresponds to the well-known phenomenon of allometry. In the case of specimens belonging to the same stage, in our case adults, we are talking of static allometry (Gould 1966). Static allometric variation might furthermore be intraspecific, i.e. amongst members of the same species or interspecific, i.e. between species (Klingenberg 2008, 2016). The nature of allometry is often similar for some species, but sometimes, it also differs in extent and direction (Rebrina et al. 2020). It is important to note that intraspecific allometry may obscure the differences in body ratios. Interspecific allometry, on the other hand, may sometimes simulate differences, where only allometric scaling, the shift along a common allometric axis is present (Gould 1966; Seifert 2002; Warton et al. 2006; Klingenberg 2008, 2016).

For a sensible interpretation of morphometric results, it is therefore essential to consider allometric variation. In many studies, such variation is simply removed from the data by various “correction” procedures (Bartels et al. 2011; Sidlauskas et al. 2011). This, for instance, is also what happens when a PCA is used in a “normal” manner. Here, the first PC comprises size, as well as all the shape variation that correlates with size, thus removing allometry from the second and all subsequent PCs (but not necessarily removing isometric size differences) (Jolicoeur 1963; Baur and Leuenberger 2011). Unfortunately, this approach does not tell us anything about the nature of allometric variation. In contrast, by applying a shape PCA within the analytical framework of MRA, allometry is not at all removed but *uncovered* by plotting shape axes (e.g. shape PCs or some body ratios) against isosize. Such plots reveal useful information about the strength and direction of allometry, which may vary between the different shape axes, as well as between groups (Mosimann 1970; Klingenberg 2016). Below, we are making use of such plots for analysing our *Tetrix* data.

We first performed a series of shape PCAs to see how well the morphs were supported by variation in shape. A shape PCA shows in very few axes (usually just the first one or two shape PCs are important) the unconstrained pattern of variation in the data. A PCA type of analysis is convenient here, as it does not require *a priori* assignment of specimens to a particular group, but assumes that all belong to a single group. We could thus avoid bias with respect to groupings (Pimentel 1979; Baur and Leuenberger 2011).

We, furthermore, employed the PCA ratio spectrum that allows an easy interpretation of shape PCs in terms of body ratios. In a PCA ratio spectrum, the eigenvector coefficients of all variables are arranged along a vertical line. Ratios calculated from variables lying at the opposite ends of the spectrum have the largest influence on a particular shape PCA; ratios from variables lying close to each other or in the middle of the graph are negligible (Baur and Leuenberger 2011; Baur et al. 2014). As usually only few variables are located at the ends, the most important variation may be spotted at a glance.

The situation changes once we specifically ask for differences *between* groups. For this question, we use a method where the groups are specified *a priori*. In the morphometry of distance measurements, such methods are usually based on linear discriminant analysis (LDA) (e.g. Hastie et al. 2009). Here, we applied a particular method of the MRA tool kit, the LDA ratio extractor (see Baur and Leuenberger 2011 for how this algorithm works). This allows the user to find the best ratio for separating two groups. Note that the algorithm not just extracts them according to discriminating power, it also ensures that successive ratios (best, second best etc.) are least correlated (Baur and Leuenberger 2011).

We used the R language and environment for statistical computing for data analysis, version 4.0.3 (R Core Team 2020). For MRA, we employed the R-scripts provided by Baur and Leuenberger (2020) on Zenodo. ANOVAs were calculated using “summary(aov())” and by using the default settings. Scatterplots were generated with the package “ggplot2” (Wickham 2016). Naturally, not all specimens in the collection were complete, which means that 95 specimens lacked one body part or another. In order to be able to include all specimens in the multivariate analyses, missing values were imputed with the R package “mice” (Buuren and Groothuis-Oudshoorn 2011), using the default settings of the function “mice()”.

Raw data in millimetres and the complete set of photographs with measurements, as well as the R-scripts used for the analyses, are available in a data repository on Zenodo (Moser and Baur 2021).

### 2) Biogeography

Given the high level of erroneous Tetrigidae determinations in collections, we refrain from incorporating published records. Instead, we concentrate on specimens studied by ourselves from several European Museums and private collections (Table 3).

**Table 3.** List of Museums and private collections with material of *bipunctata* and *kraussi* studied for the biogeography pattern. Museum codes are unified using the NCBI database (https://www.ncbi.nlm.nih.gov/biocollections/), see also Sharma et al. (2018). An exception is the Naturhistorisches Museum Bern, where we take the code used by the Museum NMBE instead of the NCBI code NHMBe.

Code	Institution
DEI	Senckenberg Deutsches Entomologisches Institut
MHNG	Muséum d'Histoire Naturelle, Geneva
MNHN	Muséum National d’Histoire Naturelle (Paris)
NMBE	Naturhistorisches Museum Bern
NHMV	Müritzeum / Naturhistorische Landessammlungen für Mecklenburg-Vorpommern
NHMW	Naturhistorisches Museum Wien
NKML	Naturkundemuseum Leipzig
SMNG	Senckenberg Museum für Naturkunde Görlitz
ZMA	Universiteit van Amsterdam, Zoologisch Museum
ZMB	Museum für Naturkunde Berlin
ZSM	Zoologische Staatssammlung München
Collectio Gatz	Katharina Gatz, Berlin, Germany
Collectio Gomboc	Stanislav Gomboc, Kranj,Slovenia
Collectio Hochkirch	Prof. Axel Hochkirch, Trier, Germany
Collectio Karle-Fendt	Alfred Karle-Fendt, Sonthofen, Germany
Collectio Landeck	Ingmar Landeck, Finsterwalde, Germany
Collectio Lehmann	Dr. Arne Lehmann, Stahnsdorf, Germany
Collectio Muth	Martin Muth, Kempten, Germany

Specimens were assigned to each morph by calculating the standard ratio (see above). After eliminating erroneous determinations by our precursors, nymphs and a single specimen of the f. macroptera which cannot be associated with either *bipunctata* or *kraussi* so far, we were able to include 660 specimens from the six Central Europe countries Germany, Netherlands, Switzerland, Austria, Italy and Slovenia (Suppl. material 1: Table S1: table of localities). Geographic coordinates and altitude were extracted from specimen labels or using standard internet sources. We analysed the biogeography stratified for *bipunctata* and *kraussi* with an emphasis on the level of sympatry and syntopy. Furthermore, we studied the altitudinal range over the north-south gradient from the northern lowlands of Germany southwards to Italy and Slovenia.

For the generation of the map, we used QGis 3.10.13-A Coruna and the Natural Earth Data (https://www.naturalearthdata.com/about/terms-of-use/, https://www.openstreetmap.org/copyright, OpenStreetMap contributors).

### 3) Microhabitat niches

In a syntopic population in Brandenburg (2.5 km E of Theisa 51.542°N, 13.503°E), the microhabitat use was studied for four months from May to August 2015 by Katharina Gatz, supervised by G.U.C. Lehmann. By slowly walking through the habitat, individuals were located either sitting or jumping from a retraceable spot. At the point of origin, a little flag was placed and the animal afterwards caught with the help of a 200 ml plastic vial (Greiner BioOne) (Fig. 8). To document the microhabitat, Katharina Gatz measured the percentage of vegetation cover and the mean vegetation height in a radius of 10 centimetres around the flag. Individuals were here also determined using the standard ratio (see above). Microhabitat niche use was available for 34 adults determined as *kraussi* and 14 *bipunctata*. Habitat data for nymphs were excluded, as the wings are not fully developed, thus preventing determination.

## Results

### Measurement data

Appendix 2 gives the descriptive statistics for each measurement (in mm) and morph as well as the sample sizes.

### Analysis using shape PCA

**Figure 2.**
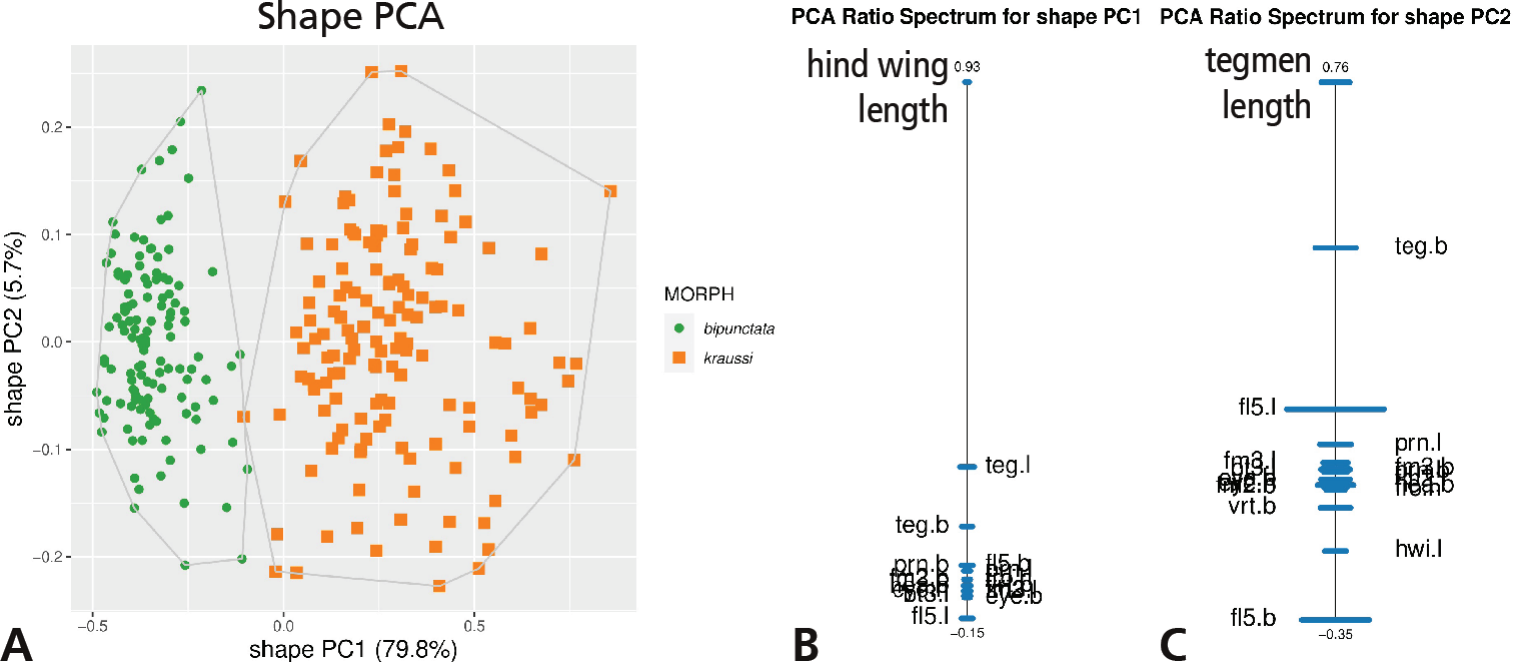
Shape principal component analysis (shape PCA) of 273 females of *Tetrix bipunctata* and *kraussi***A** analysis including 17 variables, scatterplot of first against second shape PC; in parentheses the variance explained by each shape PC **B**PCA ratio spectrum for first shape PC **C**PCA ratio spectrum for second shape PC. Horizontal bars in the ratio spectra represent 68% bootstrap confidence intervals, based on 1000 replicates; only the most important characters are indicated in ratio spectra.

We first performed a series of shape PCAs to see how well the morphs were supported by variation in shape and which body ratios were responsible for separation (Fig. 2).

In the scatterplot of the first against second shape PC, the individuals were almost perfectly separated along the first shape PC, but entirely overlapping along the second (Fig. 2A). For the interpretation of the first shape PC, we must now have a look at its PCA ratio spectrum (Fig. 2B). With this graph, we are able to read off the most important character ratios at a glance, as just those ratios are relevant that include characters lying at the opposite ends of the spectrum (in Fig. 2B, C, the only ones labelled). So, for the first shape PC, these were hind wing length (hwi.l) at the upper end and 5^th^ flagellomere length (fl5.l) at the lower end. Hence, the ratio hwi.l/fl5.l should normally be considered as the most important one. However, here the PCA ratio spectrum was noteworthy, insofar as we had, at the one end, a single character (hwi.l), whereas the other 16 characters were densely packed at the other end of the spectrum. Such an asymmetrical ratio spectrum is exceptional, since we usually observe a more symmetrical character dispersion, with few characters at the tips and the rest around the middle. Indeed, the strong asymmetry, in this particular case, profoundly influenced our interpretation. It quite simply implied that *any* ratio formed with hind wing length would result in a similar separation of the morphs! Perhaps the weakest separation should be expected from the ratio hwi.l/teg.l, because tegmen length was represented in the ratio spectrum by the bar that was a bit distant from the remaining characters at the lower end and also closest to hind wing length.

With respect to the second shape PC, the situation is quite different as there is broad overlap between *bipunctata* and *kraussi*. According to its PCA ratio spectrum (Fig. 2C), tegmen length (teg.l) to 5^th^ flagellomere breadth (fl5.b) emerged as the most important ratio. Any ratio formed with teg.l and one of the characters in the lower third of the spectrum give a similar result, as this ratio spectrum was also notably asymmetrical. Note that the overlap which we observed in morphs did not necessarily mean that none of these ratios contributed to their differentiation (see below under Extracting best ratios), but their relevance was lower. This is also reflected by the variation explained in the respective shape PCs; the first shape PC explained almost 80% of the variance, the rest less than 6% (see Fig. 2A).

### Allometry

**Figure 3.**
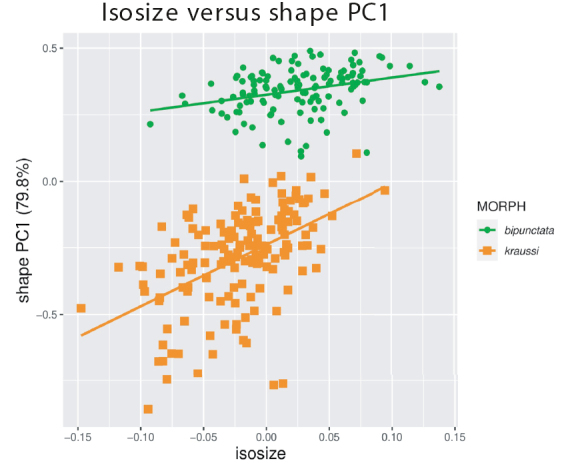
Analysis of allometric variation in 273 females of *Tetrix bipunctata* and *kraussi*. Scatterplot of isosize against first shape PC.

Plotting isosize against the first shape PC revealed that intraspecific allometry was weak in *bipunctata* and moderate in *kraussi* (Fig. 3). We were able to exclude a mere allometric scaling, because the morphs extensively overlapped in isosize, even though *bipunctata* was larger on average (ANOVA: F_1,271_ = 88.96, p < 0.001).

### Extracting best ratios

**Figure 4.**
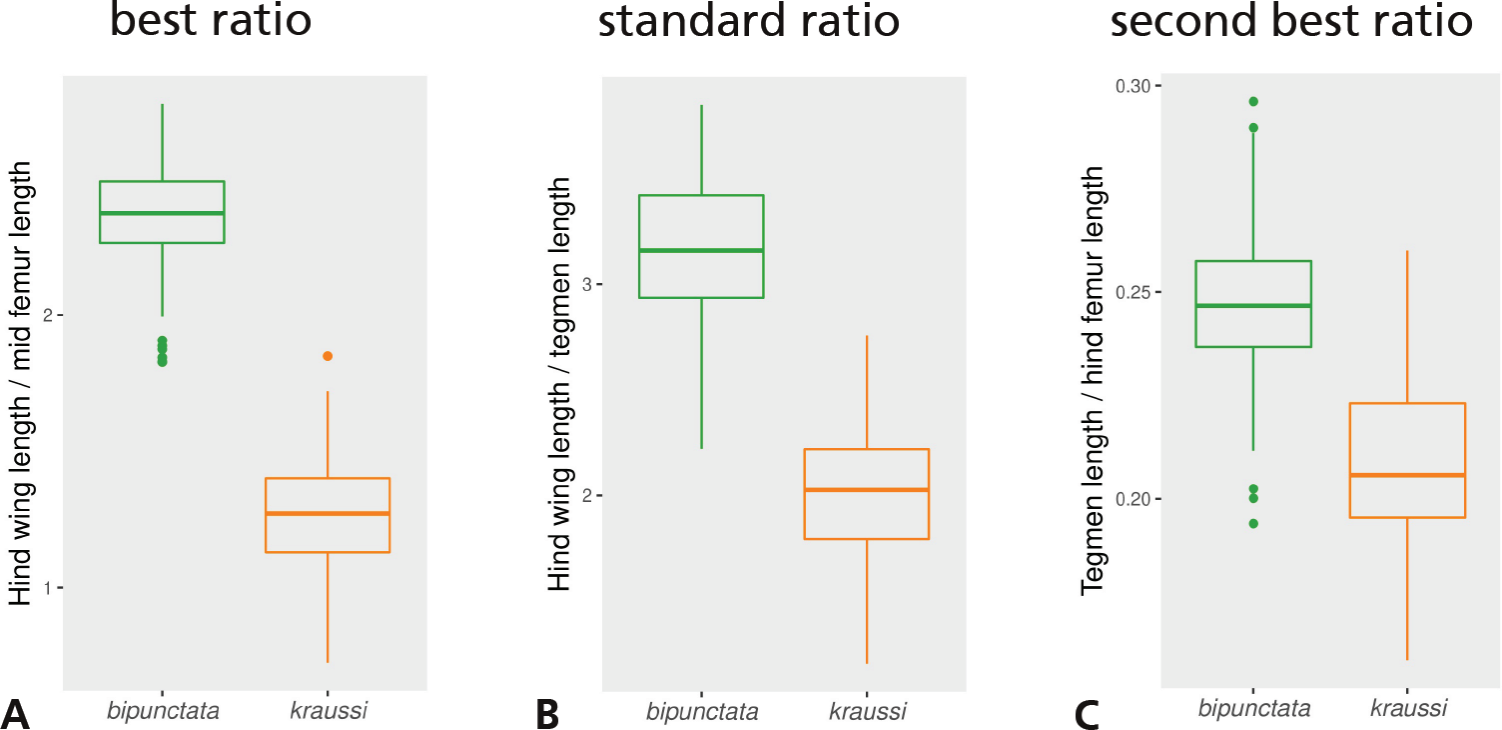
Boxplots of body ratios of 273 females of *Tetrix bipunctata* and *kraussi***A** hind wing length to mid-femur length, the ratio selected by the LDA ratio extractor as the best ratio for separating the morphs **B** hind wing length to tegmen length, the standard ratio used for discrimination **C** tegmen length to hind femur length, the second best ratio found by the LDA ratio extractor (actually the best ratio when hind wing length is omitted). Means in all plots significantly different (ANOVA, p < 0.001).

The LDA ratio extractor found hind wing length to mid-femur length as the best ratio for separating *bipunctata* from *kraussi*. This ratio was indeed more powerful than the standard ratio (compare Fig. 4A, B). In contrast, the second-best ratio found by the ratio extractor, tegmen length to hind femur length, separated the morphs much less well (Fig. 4C). However, once hind wing length was omitted, this ratio had the best discrimination power. It was also more weakly correlated with the other two ratios and thus stood for another direction in the data. This direction only revealed differences in mean (ANOVA: F_1,271_ = 795, p < 0.001), but otherwise the morphs were largely overlapping.

**Figure 5.**
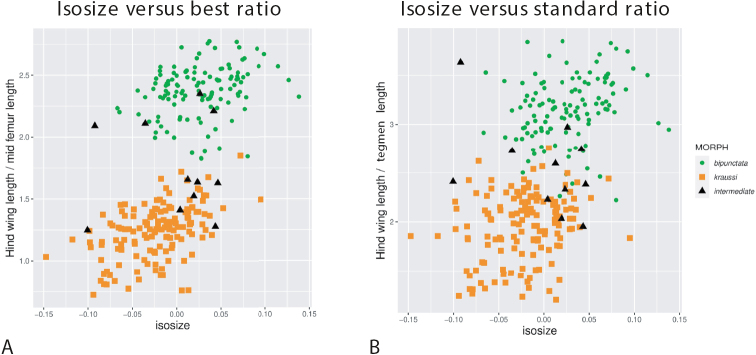
Scatterplots of isosize against body ratios of 273 females of *Tetrix bipunctata* and *kraussi*, showing the position of intermediate specimens **A** isosize against ratio of hind wing length to mid-femur length, the best ratio for separation of morphs **B** isosize against ratio of hind wing length to tegmen length, the standard ratio for discrimination (see Fig. 4). The 11 specimens considered by Nadig (1991) as “Zwischenformen” marked by black triangles.

The specimens considered as “Nadig intermediates” (“Zwischenformen”) are found in both groups. In the plot with the best ratio (Fig. 5A), these specimens were nested within each morph and, therefore, cannot be considered intermediates. In the other plot, including the standard ratio (Fig. 5B), many intermediates emerged in or near the zone of overlap.

### Biogeography

**Figure 6.**
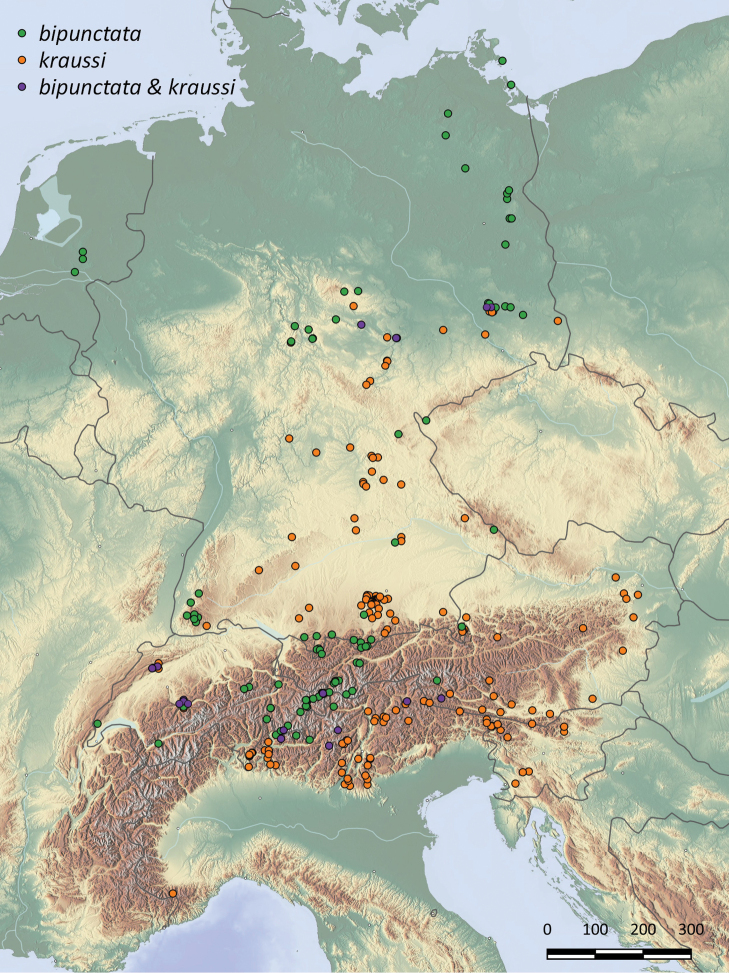
Distribution of 260 localities with records of *Tetrix bipunctata* (green dots), *kraussi* (orange dots) and syntopic populations (purple dots), mapped for six central European countries. Map generated using Natural Earth Data https://www.naturalearthdata.com/about/terms-of-use/.

**Figure 7.**
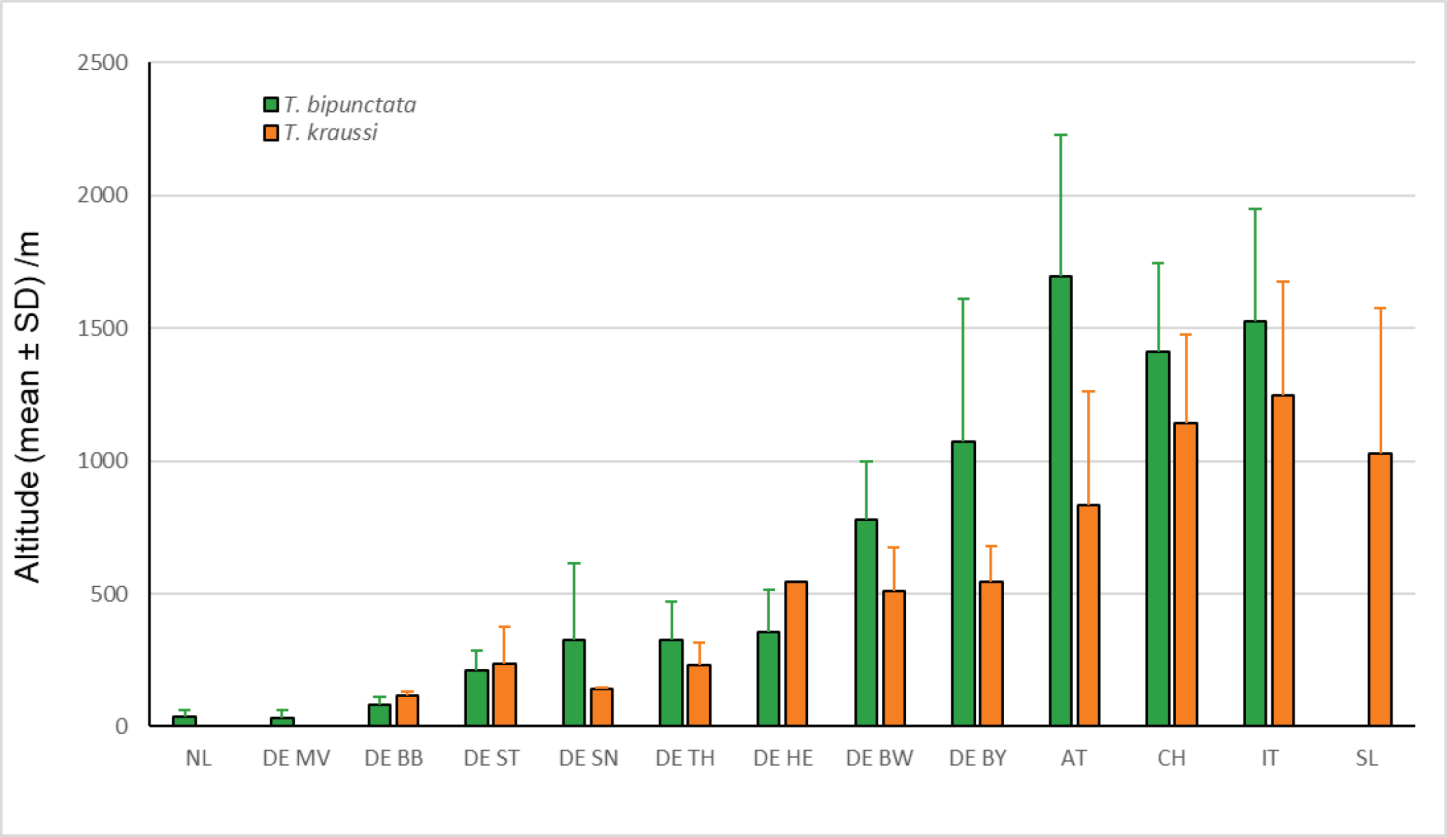
Altitudinal distribution (mean ± SD) of 286 populations of *Tetrix bipunctata* (green) and *kraussi* (orange) segmented for five Central European countries and eight Federal States in Germany. Regions are grouped along the north-south axis, NL = The Netherlands, DE = Germany: DEMV = Mecklenburg-Vorpommern, DEBB = Brandenburg, DEST = Sachsen-Anhalt, DESN = Sachsen, DETH = Thüringen, DEHE = Hessen, DEBW = Baden-Württemberg, DEBY = Bayern, AT = Austria, CH = Switzerland, IT = Italy, SL = Slovenia.

In total, 660 specimens from 286 localities could be included into our biogeographic analysis (Suppl. material 1: Table S1). We were able to include a slightly higher number of records for *kraussi*, with 403 individuals from 170 localities, than for *bipunctata* with 257 individuals from 116 localities. The general distribution pattern is largely overlapping; both *bipunctata* and *kraussi* occur in Central Europe sympatrically over much of the range (Fig. 6). However, this sympatric distribution is not perfect. In the northern lowlands area of the Netherlands, the German Federal States, Mecklenburg-Western Pomerania (Mecklenburg-Vorpommern) and ¾ of northern Brandenburg, only *bipunctata* individuals are found. All those records are below 121 m altitude, i.e. in the planar altitudinal belt. In contrast, the northernmost records of *kraussi* are from the mountainous Harz in Sachsen-Anhalt. From here, *kraussi* occurs largely sympatrically with *bipunctata* over the Central German Uplands. Given the general overlap, the low number of shared populations is notable; we identified only five syntopic localities for our German sample (two in southern Brandenburg, Thuringia Hainleite, Thuringia Kyffhäuser and Sachsen-Anhalt Balgstädt Tote Täler). In a large part of the Alps, *bipunctata* and *kraussi* are sympatric over much of their range. In Switzerland, we found syntopic populations occurring at medium altitude, especially pronounced in the Canton Bern with four out of five populations being syntopic, followed by the Jura with two out of six populations. In Beatenberg (Bernese Alps), the *bipunctata* to *kraussi* ratio was 5/9 and in Orvin (Jura), one *bipunctata* to 14 *kraussi* (Moser and Baur 2021). However, in the southern Alps, only *kraussi* occurs; all individuals from Istria up north to Carinthia (Kärnten) and Styria (Steiermark) in Austria and all pre-alpine populations in Italy, extending into the Ticino in Switzerland, belong to *kraussi*. Despite the large sympatric occurrence, a notable difference exists in the inhabited altitude. Segregated for the Federal States in Germany and the Alpine countries, *bipunctata* inhabits, on average, the higher altitudes (Fig. 7). The difference is especially clear in our samples from Austria and Bavaria, but is also found in seven out of ten regions with overlapping populations. In Slovenia, where only *kraussi* occurs, its altitudinal range is comparable to the *bipunctata* range found north of the Alps in Bavaria.

### Microhabitat niches

**Figure 8.**
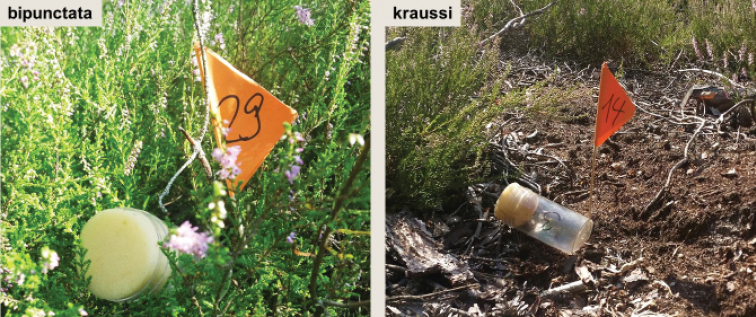
Characteristic microhabitats of *Tetrix bipunctata* (left) and *kraussi* (right) at the syntopic population at Theisa, southern Brandenburg.

In the syntopic population in Brandenburg, adults of *bipunctata* and *kraussi* show separated microhabitat niche use. While *bipunctata* adults preferentially inhabit denser vegetation with higher plants (Fig. 8a), the more open areas with less tall plants are inhabited by *kraussi* (Fig. 8b). These spots occur side-by-side in the forest aisle at Theisa in Southern Brandenburg.

**Figure 9.**
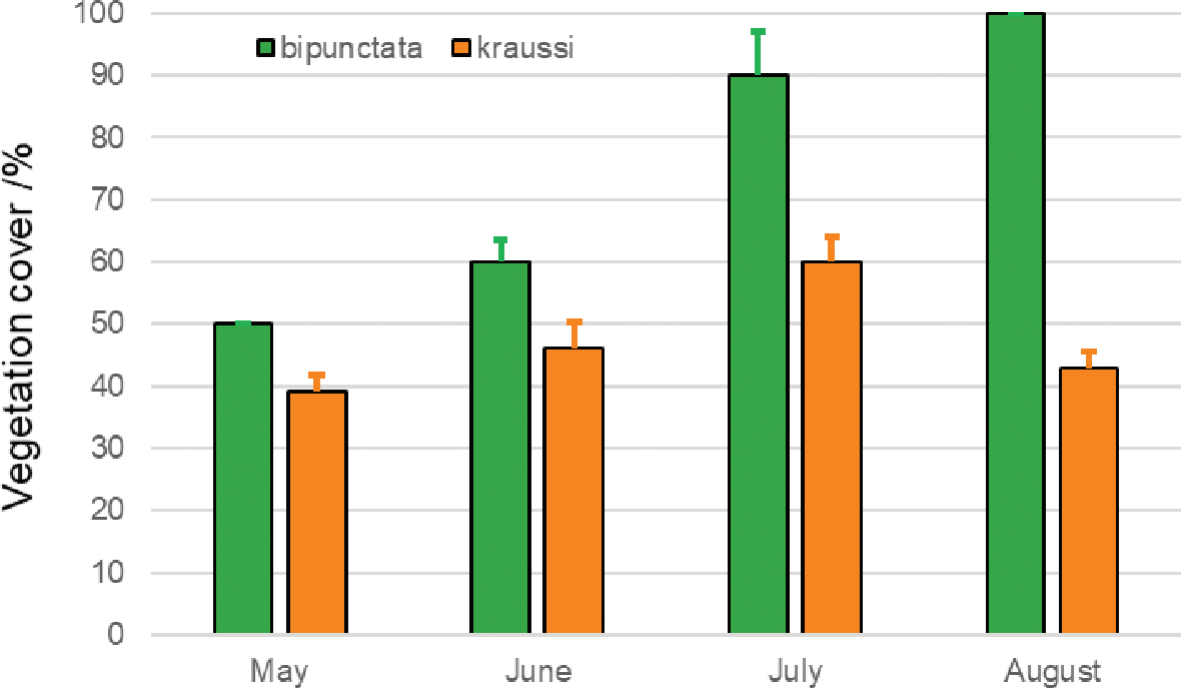
Vegetation cover in percent (mean ± SD) at spots of 10 cm diameter with records of adult *Tetrix bipunctata* and *kraussi* at the syntopic population at Theisa, southern Brandenburg.

Microhabitats of *bipunctata* had a mean vegetation cover of 70 ± 18%, nearly twice as dense as the vegetation at *kraussi* spots (40 ± 7%) (Fig. 9). This difference in vegetation cover was significant between morphs (two-way ANOVA: F_1,47_ = 455.77, p < 0.001) and between months (ANOVA: F_3,45_ = 86.33, p < 0.001). Even if the preference for more dense vegetation cover increases for *bipunctata* over the season, this shift was not significant, as indicated by the interaction term (ANOVA: F_3,45_ = 2.16, p = 0.11).

**Figure 10.**
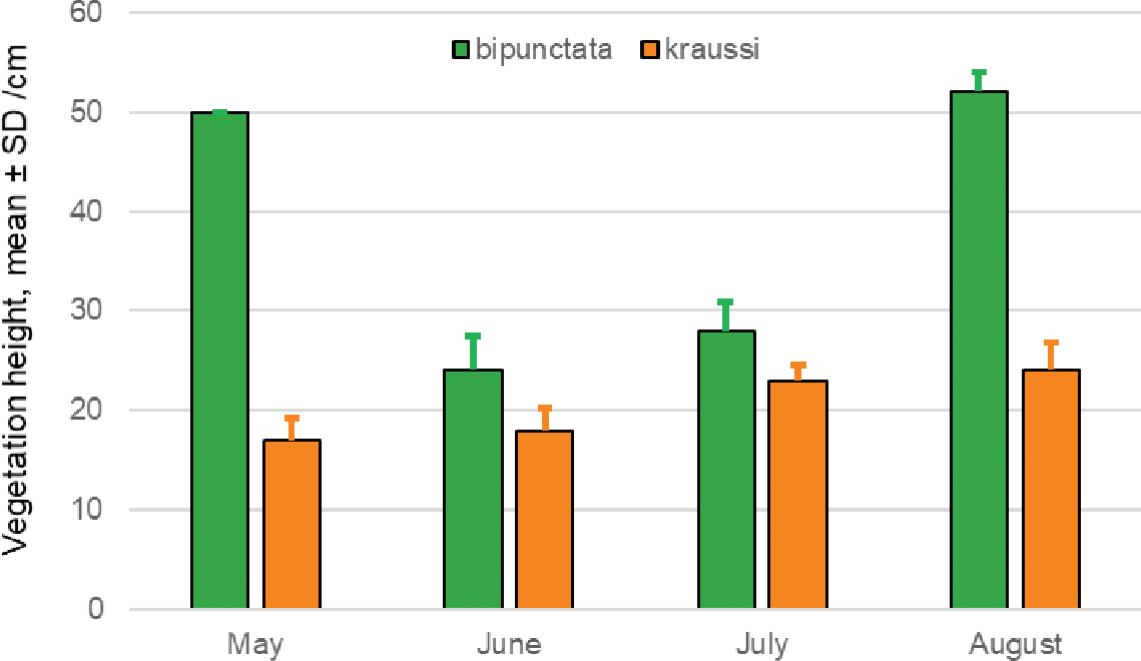
Vegetation height (mean ± SD) at spots of 10 cm diameter with records of adult *Tetrix bipunctata* and *kraussi* at the syntopic population at Theisa, southern Brandenburg.

The vegetation at sites inhabited by *bipunctata* adults was on average 27 cm ± 12 cm tall, nearly twice as high as the plants at patches with *kraussi* occurrence (16 cm ± 4 cm) (Fig. 10). The difference is significant between morphs (two-way ANOVA: F_1,47_ = 156.24, p < 0.001) and months (ANOVA: F_3,45_ = 37.80, p < 0.001). Furthermore, it was pronounced in May and August as revealed by the significant interaction term (ANOVA: F_3,45_ = 62.66, p < 0.001).

## Discussion

The morphometric analyses revealed that the morphs are merely separated by hind wing length or hind wing length in combination with any other character as a shape ratio. It was thus, by far, the most important character (Figs 2A, B, 4A, B). The first shape PC explaining 80% of the total variance supports this suggestion, while all other shape axes explain just a marginal portion of variation. The best ratio is hind wing length to length of the mid-femur, which almost perfectly distinguishes between *bipunctata* and *kraussi*. The traditionally used standard ratio of tegmen length to hind wing length (Lehmann 2004; Baur et al. 2006), is much less reliable (Fig. 4B). The differences between the morphs vanish when the importance of hind wing length is suppressed, as in the second shape PC (see Fig. 2A, C).

Isometric size between the morphs is widely overlapping with *bipunctata* being slightly larger on average (Fig. 3). This is consistent with the differences in body size measured as pronotum length and tegmen length found in the Diemeltal in northwest Germany (Schulte 2003). Based on more than 1000 specimens, *bipunctata* was the slightly larger morph compared to *kraussi.* Allometric variation is weak in both morphs and, because of the overlap in size, allometric scaling can be excluded. Some authors suggested the height of the pronotum as a possible difference (Schulte 2003). In Suppl. material 3: Fig. S2, we demonstrate that the variation in pronotum height between individuals excludes it from being a delimitation character.

In conclusion, we did find clear morphometric differences between *bipunctata* and *kraussi* only in hind wing length and all ratios including this variable. This is in agreement with results by Schulte (2003) who used specimens from northwest Germany and Sardet et al. (2015) who analysed French specimens. This means that the differences in wing length are consistent, regardless of the geographic origin.

### Nadig’s intermediate specimens and the subspecies hypothesis

Our analyis shows that the specimens from the Engadin, determined as intermediates (“Zwischenformen”) by Nadig (1991), actually fall into either the *bipunctata* or the *kraussi* cluster. This is most evident from the scatterplot of isosize versus the best ratio (Fig. 5A) and, to a lesser degree, also from isosize versus the standard ratio (Fig. 5B).

Based on his observation, Nadig (1991) proposed to classify *bipuncata* and *kraussi* as subspecies. However, the definition of a subspecies, as suggested by most authors (Mayr 1963; Mallet 2007; Braby et al. 2012), also requires a geographical separation of populations. Even though there are areas where only *kraussi* (the Southern Alps, as well as the Western Balkan) or *bipunctata* is found (Northern German Depression from the Netherlands towards Poland [this article], as well as Siberia and Scandinavia [Lehmann 2004]), there is a large area of sympatry in Central Europe (Fig. 6), thus eliminating the subspecies hypothesis. Syntopic populations are, furthermore, documented from all over the shared distribution range, with changing distribution ratio (see Schulte 2003; Lehmann and Landeck 2011; Sardet et al. 2015a; Moser and Baur 2021).

### Habitat differentiation

The morphs show a preference for slightly different habitats, with *kraussi* preferring shorter and less dense vegetation cover (Fig. 8). Fischer (1948) had already reported differential micro-habitat usage of *bipunctata* and *kraussi*, with the distribution of *bipunctata* in generally higher vegetation and less exposed than *kraussi*. We found the same in a sympatric population in Southern Brandenburg. Overall, *kraussi* seems to prefer drier, warmer climatic conditions and is often associated with limestone and open space with low vegetation, while *bipunctata* shows a preference for denser vegetation and higher plants (Figs 9, 10), which is in accordance with other observations (Zuna-Kratky et al. 2017). Where the habitat preferences overlap, the morphs meet in sympatry. These shifted preferences help to explain the altitudinal differentiation with *bipunctata* occurring at higher altitudes in the mountains (Fig. 7). Consistent with these habitat preferences, *kraussi* occurs more in the South than *bipunctata* (Fig. 6). In the syntopic populations, we recorded dominance of either *bipunctata* or *kraussi*, as reported in literature (Schulte 2003; Sardet et al. 2015; Moser and Baur 2021). This might be influenced by the prevailing climatic conditions, with *kraussi* being more common in warmer regions and *bipunctata* dominating in cooler climate.

The question whether *kraussi* and *bipunctata* represent different species or should be interpreted as infraspecific morphs is still open. The lack of genetic differentiation (see Hawlitschek et al. 2017) is equally congruent with *bipunctata* and *kraussi* being two young species or representing ecomorphs of a single species. Polymorphism, especially regarding wing length, is a well-known phenomenon in Tetrigidae, for example, in the well-studied *Tetrix subulata* (Steenman et al. 2013, 2015; Lehmann et al. 2018). To complicate the situation, a macropterous morph is documented for *bipunctata* (Devriese 1996; Schulte 2003; this study). As all known Tetrigidae are either mono- or dimorphic (e.g. Günther 1979; Devriese 1996), this would make the *bipunctata*-complex the only documented case with three wing morphs. However, this is not impossible, as other insects are able to develop several morphs per species (West-Eberhard 2003). Unfortunately, we lack any studies on the processes triggering the difference between *kraussi* and *bipunctata* and the forma macroptera as well. The mechanisms for the development of the forma macroptera, on the one hand and the switch between the morphs *bipunctata* and *kraussi* on the other hand, might differ and be based on distinct genetic backgrounds.

More research is needed to distinguish between the two possibilities that *bipunctata* and *kraussi* are genetically young species or infraspecific ecomorphs. However, this is a prime example how even modern species concepts can reach their limits. What we can exclude is their status as subspecies. Missing evidence concerns the genetic and developmental mechanisms behind the wing length. Crossing experiments could, furthermore, be informative to study reproductive barriers and hybrid disadvantage. We recommend that *bipunctata* and *kraussi* are considered as separate units until the species question can be answered more precisely.

## Appendix 1

Identification and removal of unreliable characters.

As mentioned under Materials and methods, we omitted three characters from all morphometric analyses presented in the results. In the following, we briefly describe the procedure that led to their removal.

Initially, we started with a shape PCA, based on all 20 characters (see Suppl. material 2: Fig. S1). The resulting scatterplot was very similar to the one presented in the results (Fig. 2A), with an almost perfect separation of morphs along shape PC1 and a complete overlap along shape PC2 (Suppl. material 2: Fig. S1A). In addition, the PCA ratio spectrum for shape PC1 was fully congruent with the one of the definitive analysis (compare Suppl. material 2: Fig. S1B and Suppl. material 3: Fig. S2B). Differences eventually arose in the PCA ratio spectrum of the second shape PC, where the coefficients of the three characters pronotum height (prn.h), 2^nd^ pulvillus length (pu2.l) and 3^rd^ pulvillus length (pu3.l) evidently had much too broad confidence intervals (Suppl. material 2: Fig. S1C). These characters dominated the spectrum (also that of the third shape PC, not shown here), but at the same time, did not at all contribute to the differentiation of morphs. We, therefore, suspected that the measurements were unreliable, either due to high measurement error or intraspecific variation (Baur and Leuenberger 2011). Closer inspection of specimens, indeed, revealed that the latter was prevalent concerning the upper edge of the pronotum. Here, specimens of both morphs showed large individual variation. For measuring pronotum height, we thus had to move the reference points along the body axis, rendering these points clearly non-homologous (Suppl. material 3: Fig. S2, measurement position indicated by a magenta line; note the varying position of these lines relative to the base of the tegmen). The pulvilli, on the other hand, were often worn off and the respective reference points indistinct.

It is well known that a high quality of measurements is crucial in morphometric data, as low reliability may cause serious problems for multivariate data analysis (Lougheed et al. 1991; Bartlett and Frost 2008; Nakagawa and Schielzeth 2010; László et al. 2013). Baur et al. (2014), for instance, demonstrated how badly a single error-prone variable may affect a shape PCA by masking important groupings. Therefore, we think it was not only justified, but also necessary to exclude the three characters from the dataset.

## Appendix 2

Overview of measurements of *Tetrix* females, showing minimum, mean, median and maximum in mm.

## Supplementary materials

Supplementary material 1 Table S1 Data type table Explanation note Records of 660 specimen of *Tetrix bipunctata* and *T. kraussi*, based on our surveys in European Museums and private collections, see Table 3 for the list of sources. The 17 rows coloured represent syntopic occurrences of *Tetrix bipunctata* and *T. kraussi*. Species z = Zwischenformen, specimen supposed to be intermediates by Nadig (1991), but turned out to be either *Tetrix bipunctata* or *T. kraussi* in this study. Date Collection date as reported on labels, in square brackets we added the unreported centuries [18] or [19] deduced from our knowledge of collectors biographies. State English name of the governmental province. Bundesland / Kanton: German name of the governmental province. Geographic coordinates and altitudes extracted with the help of open mapping tools (https://tools.retorte.ch/map/, https://www.mapcoordinates.net). Comments Additional information given on labels. First and second determination Identifications based on label information. Authors’ determination: Identifications based on the standard ratio of the full hind wing length to tegmen length: ≥ 2.5 = bipunctata, < 2.5 = kraussi (corresponding to the ratio of the protruding part of hind wing length to tegmen length of ≥ 1.5 and < 1.5, respectively). Collectio Abbreviations of European Museums and private collections with material studied. Museum codes are unified using the NCBI database (https://www.ncbi.nlm.nih. gov/biocollections/), see also Sharma et al. (2018). An exception is the Naturhistorisches Museum Bern, where we take the code used by the Museum NMBE instead of the NCBI code NHMBe (compare Table 3). Collection number Individual codes assigned by the Collectio Lehmann [CL], the Muséum d'Histoire Naturelle, Geneva (MHNG) or Naturhistorisches Museum Bern (NMBE). https://binary.pensoft.net/file/554316This dataset is made available under the Open Database License (http://opendatacommons.org/licenses/odbl/1.0/). The Open Database License (ODbL) is a license agreement intended to allow users to freely share, modify, and use this Dataset while maintaining this same freedom for others, provided that the original source and author(s) are credited.Valentin Moser, Hannes Baur, Arne W. Lehmann, Gerlind U.C. Lehmann

Supplementary material 2 Figure S1 Data type (measurement/occurrence/multimedia/etc.) Explanation note Shape principal component analysis (shape PCA) of 273 females of *Tetrix bipunctata* and *kraussi*. **A**: analysis including 20 variables, scatterplot of first against second shape PC. **B**: PCA ratio spectrum for first shape PC. **C**: PCA ratio spectrum for second shape PC. Horizontal bars in the ratio spectra represent 68% bootstrap confidence intervals based on 1000 replicates. https://binary.pensoft.net/file/554317This dataset is made available under the Open Database License (http://opendatacommons.org/licenses/odbl/1.0/). The Open Database License (ODbL) is a license agreement intended to allow users to freely share, modify, and use this Dataset while maintaining this same freedom for others, provided that the original source and author(s) are credited.Valentin Moser, Hannes Baur, Arne W. Lehmann, Gerlind U.C. Lehmann

Supplementary material 3 Figure S2 Data type (measurement/occurrence/multimedia/etc.) Explanation note Variation in pronotum shape (lateral view) of some *Tetrix* females included in the morphometric analyses. **A–D**: *bipunctata*; **E–H**: kraussi. The position where pronotum height was measured is indicated by a magenta line. https://binary.pensoft.net/file/554318This dataset is made available under the Open Database License (http://opendatacommons.org/licenses/odbl/1.0/). The Open Database License (ODbL) is a license agreement intended to allow users to freely share, modify, and use this Dataset while maintaining this same freedom for others, provided that the original source and author(s) are credited.Valentin Moser, Hannes Baur, Arne W. Lehmann, Gerlind U.C. Lehmann
