# Supplementary material for: Two species? – Limits of the species concepts in the pygmy grasshoppers of the Tetrix bipunctata complex (Orthoptera, Tetrigidae)
Source: Zookeys. 2021 Jun 11;1043:33–59. doi: 10.3897/zookeys.1043.68316 (PMC8213684; doi:10.3897/zookeys.1043.68316)
Supplement: Supplementary material 2 — Figure S1 [file zookeys-1043-033-s002.pdf]

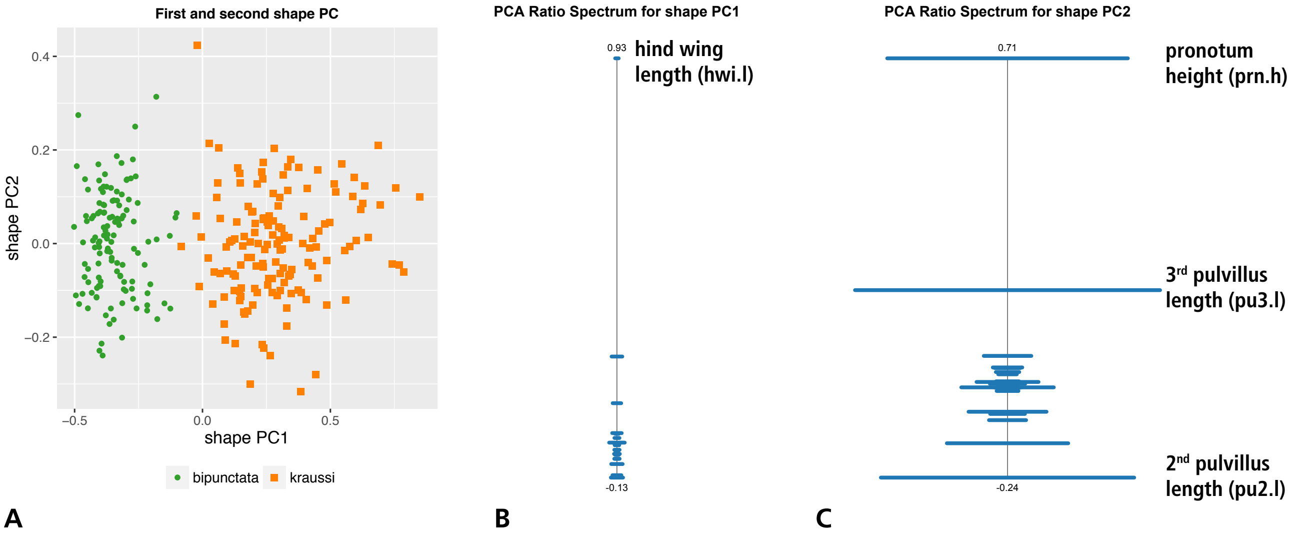

Supplementary Figure 1. Shape principal component analysis (shape PCA) of 273 females of *Tetrix bipunctata* and *kraussi*. **A**: analysis including 20 variables, scatterplot of first against second shape PC. **B:** PCA ratio spectrum for first shape PC. **C:** PCA ratio spectrum for second shape PC. Horizontal bars in the ratio spectra represent 68% bootstrap confidence intervals based on 1000 replicates.
