# Supplementary material for: Two species? – Limits of the species concepts in the pygmy grasshoppers of the Tetrix bipunctata complex (Orthoptera, Tetrigidae)
Source: Zookeys. 2021 Jun 11;1043:33–59. doi: 10.3897/zookeys.1043.68316 (PMC8213684; doi:10.3897/zookeys.1043.68316)
Supplement: Supplementary material 3 — Figure S2 [file zookeys-1043-033-s003.pdf]

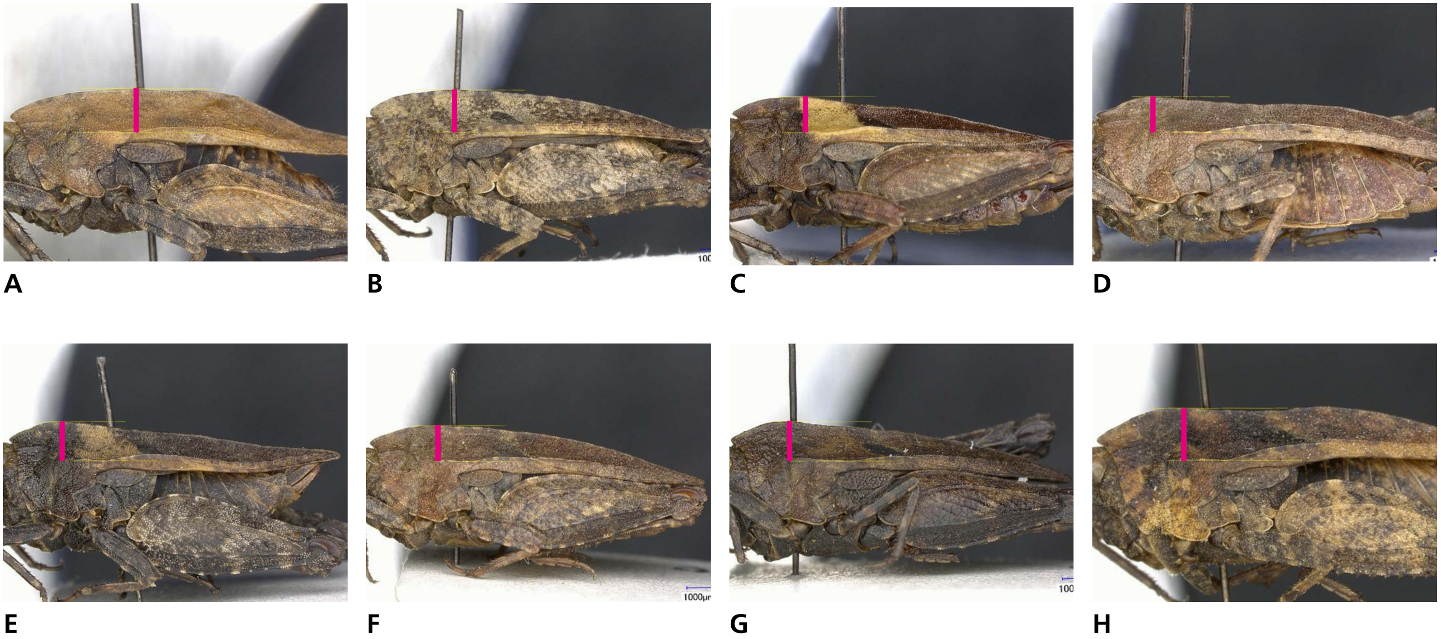

Supplementary Figure 2. Variation in pronotum shape (lateral view) of some *Tetrix* females included in the morphometric analyses. **A–D:** *bipunctata*; **E–H:** *kraussi*. The position where pronotum height was measured is indicated by a magenta line.
